# Effects of an Impaired Fasting Glucose on the Left Atrial Strain Evaluated by Speckle Tracking Echocardiography

**DOI:** 10.3390/medicina59111982

**Published:** 2023-11-10

**Authors:** Gülsüm Bingöl, Fulya Avcı Demir, Emre Özmen, Serkan Ünlü, Özge Özden, Kardelen Ohtaroğlu Tokdil, Leyla Bulut Arsoy, Fatma Özpamuk Karadeniz, Barış Ökçün

**Affiliations:** 1 Department of Cardiology, Istanbul Arel University, 34537 Istanbul, Turkey; bulut_gulsum@hotmail.com; 2 Department of Cardiology, Antalya Medical Park Hospital, 07160 Antalya, Turkey; fulyaavcidemir@gmail.com; 3 Memorial Bahçelievler Hospital, 34180 Istanbul, Turkey; dremreozmen@yahoo.com (E.Ö.); ozgeozdenctf@hotmail.com (Ö.Ö.); dr_baris_2000@yahoo.com (B.Ö.); 4 Department of Cardiology, Gazi University Faculty of Medicine, 06570 Ankara, Turkey; unlu.serkan@gmail.com; 5 Department of Cardiology, Zonguldak Devrek Hospital, 67800 Zonguldak, Turkey; kardelenohtaroglu@gmail.com; 6 Department of Biochemistry, İstanbul Göztepe Prof Dr. Süleyman Yalçın City Hospital, 34722 İstanbul, Turkey; leylabulut_83@hotmail.com; 7 Department of Cardiology, Medical Faculty, Karamanoğlu Mehmetbey University, 70100 Karaman, Turkey

**Keywords:** strain echocardiography, diabetes mellitus, fasting blood glucose

## Abstract

*Background and Objectives*: Similar to diabetes, the presence of left ventricular (LV) diastolic function (DD) has been reported in various studies which were conducted with people with a diagnosis of an impaired fasting blood glucose (FBG). This study aimed to examine the effects of the fasting blood glucose (FBG) levels on the left atrial strain (LAS) estimated by two-dimensional echocardiography speckle tracking analyses in patients without known diabetes. *Material and Methods*: The study included 148 participants (74 female and 74 male) without a history of diabetes mellitus or chronic disease. The patients were divided into two groups as follows: individuals with an FBG < 100 mg/dL and those with an FBG between 100 and 125 mg/dL after at least 8 h of overnight fasting. According to these FBG levels, speckle tracking echocardiography (STE) measures were compared. *Results*: There was a significant decrease in the LA reservoir (52.3 ± 15 vs. 44.5 ± 10.7; *p* = 0.001) and conduit strain (36.9 ± 11.7 vs. 28.4 ± 9.7; *p* = 0.001) in the impaired FBG group. When the STE findings of both ventricles were compared, no significant difference was observed between the groups in right and left ventricular strain imaging. *Conclusions*: In the earliest stage of LVDD, changes in atrial functional parameters become particularly evident. Echocardiographic analyses of these parameters can help to diagnose and determine the degree of LVDD while the morphological parameters are still normal. The addition of LAS imaging to routine transthoracic echocardiography (TTE) studies in patients with an impaired FBG but without a DM diagnosis may be helpful in demonstrating subclinical LVDD or identifying patients at risk for LVDD in this patient group.

## 1. Introduction

Diabetes mellitus (DM) significantly increases the risk of developing cardiovascular disease (CVD) [1]. While the American Diabetes Association (ADA) defines DM as having a fasting blood glucose (FBG) of ≥126 mg/dL or an HbA1c value of ≥ 6.5%, it has classified people with an FBG between 100 and 125 as having prediabetes [2]. Similar to DM, it has also been shown that prediabetes, which is also called impaired FBG and is considered to be part of the onset of DM, increases the risk of CVD [1]. Prediabetes is characterised by an elevated risk of cardiovascular disease (CVD) due to factors such as higher endothelial dysfunction, a high atherogenic lipid profile, a higher incidence of hypertension, and increased indications of a pro-thrombotic condition.

One of the cardiovascular diseases caused by an impaired FBG is heart failure (HF) [3]. The relationship between impaired left ventricular (LV) diastolic function (DD) parameters and an impaired FBG, which is one of the earliest changes that leads to this condition, has been proven in several previous studies [4,5]. This precedes systolic dysfunction and associates HF with a reduced ejection fraction (EF) [4].

The left atrial strain (LAS) is a new parameter that is used in the evaluation of the LV filling pressure and the LV diastolic function [6,7]. The LAS comprises three phases as follows: reservoir (LAS-r), conduit (LAS-cd) and contractile (LAS-ct). The LAS in the reservoir phase corresponds to early diastole in the LA, marked by the maximum relaxation of its wall and commonly known as Peak Atrial Longitudinal Strain (PALS). The LAS-cd aligns with LA mid-diastolic emptying through the passive shortening of its wall. In the LAS-ct or Peak Atrial Contraction Strain (PACS), the LA synchronises with the LA systole, actively shortening the myocardium and contributing to LV filling [8]. In cases of normal diastolic function, the relative contributions of these distinct LA phases to LV filling are as follows: the reservoir phase contributes 40%, the conduit phase 35%, and the contractile phase 25% [9]. The LAS, which provides important prognostic information in various cardiac pathologies, instantly reflects the left atrial (LA) pressure and reveals prognosis better than the LA volume index (LAVI) [7,10,11].

Although diastolic function studies in patients with an impaired FBG are generally conducted with LV diastolic function parameters, we planned to investigate whether there is a difference in LAS findings between individuals with a normal FBG and an impaired FBG without known diabetes in our study.

## 2. Methods

### 2.1. Study Population

This was a single-centre, retrospective study of echocardiographic measures of LV diastolic function and LAS. A total of 148 individuals without any known chronic disease who were admitted to our cardiology outpatient clinic between January 2021 and January 2022 were included in the study. The enrolment of patients with an absence of chronic disease and verified absence of any chronic medication history was corroborated through patient anamnesis and a national database. The patients were divided into two groups: those with an FBG between 70 and 100 mg/dL and those with an FBG between 100 and 125 mg/dL after at least 8 h of overnight fasting. The criterion for an impaired FBG was determined according to the criteria of the ADA [2]. The study was approved by the local ethics committee of our own institution. The study was conducted according to the guidelines of the Declaration of Helsinki and of the local ethics committee of our own institution. 

The physical examination findings, electrocardiography and transthoracic echocardiography (TTE) data, and laboratory values of the patients included in the study were obtained from the hospital database. Those with an FBG < 70 mg/dL or >125 mg/dL and those whose echocardiographic images were insufficient for speckle tracking analyses were excluded from the study. Patients with valvular disease, pulmonary hypertension, left or right ventricular dysfunction on echocardiography, symptoms or signs of cardiovascular disease, arterial hypertension, heart failure, previous myocardial infarction, moderate or severe valvular disease, arrhythmias, congenital heart diseases, asthma, chronic obstructive lung disease, neoplastic disease, morbid obesity, cirrhosis, or renal failure were excluded from the study.

### 2.2. Echocardiographic Assessment

TTE examinations were performed by an experienced cardiologist with a Philips Epiq 7C ultrasound machine according to the recommendations of the American Society of Echocardiography [12]. All echocardiography examinations included standard M-mode images, two-dimensional imaging, tissue Doppler evaluation at the septal and lateral mitral annulus and strain imaging in all patients at rest in the left decubitus position.

LVEF was calculated by using the standard Simpson method. The transmitral Doppler inflow and tissue pulsed Doppler velocities were obtained in the apical 4-chamber view. Pulsed Doppler measurements included the transmitral early and late diastolic peak flow velocities (Em and Am) and their ratio (E/Am). The average of the peak early diastolic relaxation velocities (e′) of the septal and lateral mitral annulus was calculated.

### 2.3. Strain Analysis

**Figure 1:**
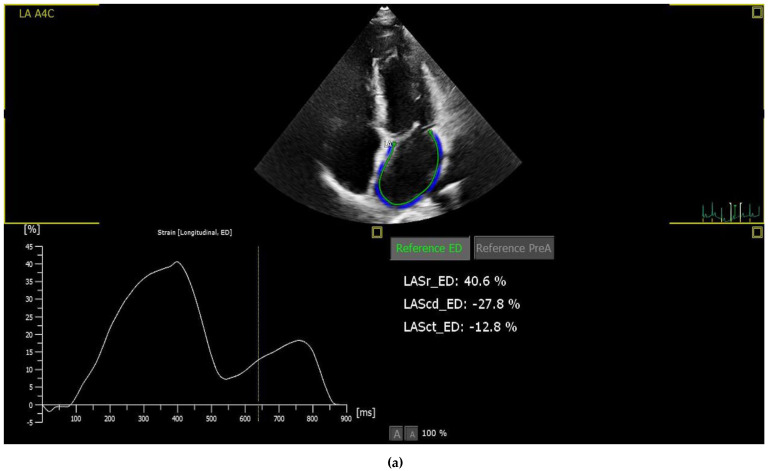

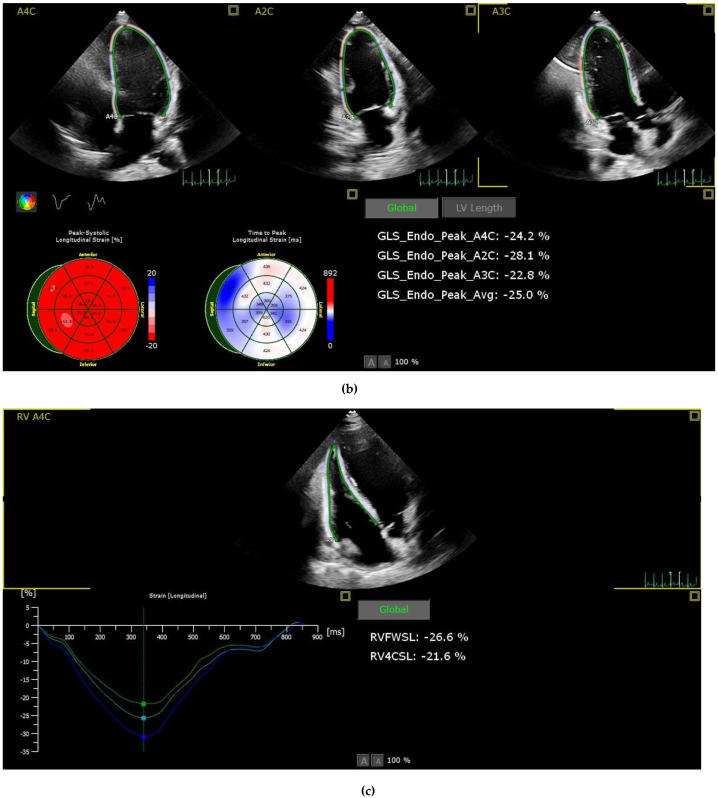
Imaging of STE components of left atrial (**a**), left ventricle (**b**) and right ventricle strain (**c**). **Abbreviations:** LA: Left atrium, A4C: Apikal four chamber, LASr: Left atrial reservoir strain, LAScd: left atrium conduit strain, LASct: left atrium contractile strain, A2C:Apikal two chamber, A3C:Apikal three chamber, GLS: Global longitudinal strain, Avg: Average, LV: Left ventricle, RVFWSL: Right ventricle free wall strain longitudinal, RV4CSL: Right ventricle four chamber strain longitudinal.

Patients with suboptimal image quality for strain analysis were excluded from the study. The echocardiographic images were analysed by a cardiologist who is particularly interested and trained in cardiovascular imaging and strain echocardiography. The strain values, including the right ventricle GLS (RVGLS) and right ventricle free wall strain (RV-FWSL), the LVGLS, and components of the LAS (atrial reservoir (LAS-r), conduit (LAS-cd), and contractile (LAS-ct) function), were examined from the stored echocardiographic images using a commercially available workstation (QLAB version 13) and by following the recommendations of the EACVI [13] (Figure 1).

Speckle tracking echocardiography (STE) was performed using four consecutive cardiac cycles of two-dimensional LV images from the three standard apical LV, LA, and RV focused apical 4-chamber views, according to the latest guidelines [13]. For strain analysis, novel 2D strain analytical software (AutoStrain Qlab13, Philips health systems, Netherlands) was used that automatically defines the region for tracking by forming a line around the region while still allowing manual changes afterward. The tracking quality was checked by comparing the motion of the endocardium by the investigator. Manual adjustments were performed after a visual assessment of the tracking results throughout the entire cardiac cycle. In segments with poor tracking results, the border was readjusted manually until the best tracking was provided.

### 2.4. Laboratory Analysis

All participants were interviewed at each annual examination using standard questionnaires that collect data on demographic characteristics and medical history. Blood samples were then taken after a 12 h fast and analysed immediately. The IFG was defined according to current guidelines [2] as a 12 h fasting plasma glucose measurement between 100 and 125 in subjects without known DM. Together with the FPG, the complete blood count, renal function panel, total cholesterol, low density lipoprotein cholesterol (LDL-C), HDL-C, and thyroid stimulant hormone (TSH) levels were evaluated.

### 2.5. Statistical Analysis

All statistical analyses were performed using SPSS v25.0. Continuous variables are presented as the mean and standard deviation, and categorical data are presented as percentages or frequencies. A Kolmogorov–Smirnov test was used to determine the normality of the distribution of continuous variables. Parametric and nonparametric variables were compared by using paired t tests and Wilcoxon tests, respectively. Categorical variables were compared by using a chi-square (χ^2^) test. The patient population was divided into two groups according to blood glucose levels. A univariate linear regression analysis and a multivariate linear regression model were tested for determining LAS-r by entering conventional clinical variables (age, BMI) and contributors with a significant correlation. Results for which the *p* value was <0.05 were considered statistically significant.

## 3. Results

The study included 148 subjects (74 female and 74 male). The demographic, clinical, and laboratory characteristics of the patients included are given in Table 1.

When the normal FBG group was compared with the impaired FBG group, the mean age of the impaired FBG group was higher (33.2 ± 9 vs. 38.5 ± 10.1; *p* = 0.001), and no significant difference was found in other demographic parameters (Table 1). No significant difference was observed in other laboratory parameters (Table 1). There were significant differences between the groups in several markers of LV diastolic function.

Considering all TTE parameters, the LA was larger (3.1 ± 0.5 vs. 3.3 ± 0.4; *p* = 0.036), the LA volume was greater (39.7 ± 16 vs. 48.1 ± 17.8; *p* = 0.006), the right atrium was larger (3.3 ± 0.5 vs. 3.4 ± 0.5; *p* = 0.05), the IVRT was longer (82.7 ± 19.9 vs. 93.4 ± 22.6; *p* = 0.005), the mitral E wave was shorter (0.8 ± 0.2 vs. 0.7 ± 0.2; *p* = 0.037), and the septal e’ was lower (10.8 ± 2.1 vs. 9.6 ± 2.2; *p* = 0.001) in the impaired FBG group.

When the STE findings of both ventricles were compared, no significant difference was observed between the groups in right and LV strain images. A comparison of the STE findings of the groups is summarised in Table 2.

### Left Atrium Function Differences between the Groups

While there was a significant decrease in the LA reservoir (52.3 ± 15 vs. 44.5 ± 10.7; *p* = 0.001) and conduit strain (36.9 ± 11.7 vs. 28.4 ± 9.7; *p* < 0.001) in the impaired FBG group, no significant difference was observed in contractile strain (15.4 ± 9.4 vs. 16.1 ± 7.3; *p* = 0.653). The findings from the LA speckle tracking imaging of the patients are shown in Table 2. The determinants of LAS-r by multivariate analysis are shown in Table 3.

## 4. Discussion

In this study, we aimed to evaluate the effect of an impaired FBG, one of the most important parameters of metabolic syndrome, on the LA strain, which is a reliable measure for evaluating LA functions.

The main findings of our study can be listed as follows: 1. The LAS-r and LAS-cd strain values were found to be lower in the patient group with an impaired FBG than in the normal FBG group. 2. The left and right atrial diameters and LA volume were found to be larger in the group with an impaired FBG. These results show that LAS values are more decreased in the group of patients with an impaired FBG than in the normal FBG group. In accordance with multivariate analysis, LVGLS, LVEF, and age exert an influence, but it is noteworthy that LAS-r demonstrates the most significant *p*-value in the determination of serum fasting glucose levels.

Various studies have shown that echocardiographic LV diastolic function and strain imaging parameters are impaired in patients with DM compared to individuals without a diagnosis of DM [14,15], and the prevalence of LVDD in patients with DM is significantly higher than that in the general population [16]. The aforementioned findings are of the utmost importance, as they are associated with one of the most critical risk factors for the development of atrial fibrillation and heart failure with preserved ejection fraction (HFpEF) [17,18]. Both of those conditions are associated with global and cardiovascular hospitalisation and mortality [19].

Similar to diabetes, the presence of LVDD has been reported in various studies that were conducted with people with a diagnosis of impaired FBG [4,20].

In a study conducted on 2971 middle-aged people, Milwidsky et al. [4] observed that an impaired FBG was associated with LVDD. In that study, the presence of LVDD was determined by 2D TTE and Doppler echocardiography findings, and strain imaging was not utilised. As a result of the study, it was demonstrated that people with an impaired FBG were 43% more likely to have LVDD than people with a normal FBG. Even after considering factors like obesity, a high blood pressure, and a thickened left ventricle, an impaired fasting glucose (IFG) remains closely associated with problems in the heart’s diastolic function. In other words, even in healthy middle-aged adults without other health issues like a high blood pressure, an IFG could signal early heart problems. This link is strongest in young individuals with a normal blood pressure similar to the patient population in our study [4].

Pareek et al. [21], on the other hand, showed that echocardiographic LVDD was observed significantly more often in patients with an impaired FBG who had no comorbidities at the beginning of the study as a result of a median follow-up of 8.3 years. Similarly, in that study, diastolic function was evaluated with 2D TTE and transmitral Doppler and tissue Doppler parameters [20].

As seen in the studies above, LVDD in people with an impaired FBG is evaluated with TTE and generally by transmitral Doppler and tissue Doppler imaging.

In the earliest stage of LVDD, changes in atrial functional parameters become particularly evident. Echocardiographic analyses of these parameters can help to diagnose and determine the degree of LVDD, while the morphological parameters are still normal [7,22].

The anterior–posterior (AP) LA diameter or LA volume obtained by two-dimensional (2D) echocardiography are the simplest markers of LA remodelling [23]. Due to their simplicity and reproducibility, these markers are widely used in many studies. The LA is directly exposed to LV filling pressure during diastole; thus, LA dilation is one of the strongest indicators of LVDD [24].

In our study, the LA diameter and volume were found to be significantly higher in patients with an impaired FBG, in parallel with other studies. Diastolic dysfunction causes various changes in the functions of the LA apart from geometric changes in the LA. Echocardiographic evaluation of LA deformation is a relatively novel modality for the assessment of LA remodelling. There are two methods of assessment: tissue Doppler imaging and speckle tracking imaging. Tissue Doppler imaging is widely available and practical for routine echocardiography. The A′ velocity at the mitral annulus provides information about the regional atrial systolic motion. Early systolic (S′) and early diastolic (E′) velocities correspond to the LA reservoir and conduit function, respectively. However, these velocity measurements also bear some limitations related to angle dependency and tethering [24]. LA deformation evaluated by 2D STE imaging is generally known LA strain. Today, LAS measurements using STE are considered the most specific and sensitive method for the functional assessment of the LA [25]. LA strain was shown to be useful in the detection and grading of HFpEF. Cardiac magnetic resonance (CMR) can be used to measure the strain in the LA. However, it is important to know that using CMR for this purpose is time-consuming, expensive, and requires specialised training, making it impractical for everyday clinical use [26]. 

Studies have shown that LAS imaging is a useful additional method for evaluating the LV diastolic function [27,28]. E/e′ is an echocardiographic parameter that is a good indicator of the left ventricular diastolic function. The LA GLS has been shown to be better at predicting the LV end-diastolic pressure than the mean E/e′ ratio in a study by Cameli et al. [27]. In our study group, although there was no difference between the two groups in terms of E/e’, it was found as slightly higher in the group with a high fasting blood glucose. This demonstrated that the LAS is a more sensitive echocardiographic parameter in demonstrating left ventricular diastolic functions compared to E/e′ that presents changes early. In a study by Morris et al. [28], it was shown that the addition of LA longitudinal peak strain to the LV volume index in evaluating LVDD in patients with a preserved EF was better than the LAVI alone in determining diastolic dysfunction. In addition, it was shown that the risk of hospitalisation for HF was increased in patients with an abnormal LAS, even if the LAVI was normal in two years of follow-up [28].

Similar to diabetes, prediabetes is also associated with HF and atrial fibrillation, which are determinants of cardiovascular mortality. In a prospective study involving 294,057 patients, high glucose levels, including in the impaired FBG range, were found to be associated with the development of atrial fibrillation and HF during an average of 19.1 years of follow-up of patients with known diabetes mellitus, newly diagnosed diabetes or an impaired fasting glucose [29]. One of the possible mechanisms leading to this condition is increased fibrosis and atrial remodelling [30]. The development of LVDD or HFpEF, elevated filling pressures and volume overload of the LA could also be potential drivers for the association between dysglycaemic conditions.

The limitations of our study include the single-centre nature of the study and the small number of patients.

## 5. Conclusions

In conclusion, the addition of LAS imaging to routine TTE studies in patients with an impaired FBG but without a DM diagnosis may be helpful in demonstrating subclinical LVDD or identifying patients at risk for LVDD in this patient group. We believe that further studies are needed on this subject.

## Figures and Tables

**Table 1:** Clinical and basic biochemical features of patient groups.

	FBG 70–100 (*n* = 95)	FBG 100–125 (*n* = 53)	*p* Value
Age (year)	33.2 ± 9	38.5 ± 10.1	0.001
Gender (f, %)	45 (47.4)	29 (54.7)	0.493
Height (cm)	168.3 ± 9.3	168.9 ± 10.1	0.697
Weight (kg)	71.1 ± 13.4	74.9 ± 13.3	0.089
Body mass index (kg/m^2^)	25.01 ± 3.7	26.24 ±3.7	0.058
Current smoker (*n*, %)	41 (43.1)	18 (51.4)	0.273
Serum fasting glucose (mg/dL)	89.6 ± 14.4	110.8 ± 15.8	<0.001
Haemoglobin (g/dL)	14 ± 2.2	14 ± 1.8	0.928
WBC count (10^3^ μL)	7.4 ± 1.7	7.3 ± 1.7	0.625
Creatinine (mg/dL)	0.8 ± 0.2	0.8 ± 0.2	0.222
GFR (mL/min per 1.73 m^2^)	108.1 ± 18.6	108.2 ± 19.5	0.986
ALT (U/L)	19.6 ± 18.7	17.7 ± 7.9	0.469
AST (U/L)	20.3 ± 23.2	20.4 ± 19.2	0.975
Total cholesterol (mg/dL)	173 ± 35.8	180.4 ± 32.5	0.207
LDL cholesterol (mg/dL)	103.5 ± 32.2	110 ± 26	0.201
HDL cholesterol (mg/dL)	47.5 ± 13.3	45.6 ± 11.1	0.372
Triglyceride (mg/dL)	112.7 ± 63	124 ± 72.2	0.316
TSH (mU/L)	2 ± 1.2	2.1 ± 1.1	0.599

**Abbreviations:** FBG: fasting blood glucose, WBC: white blood cell, GFR: glomerular filtration rate, ALT: alanine transaminase, AST: aspartate transaminase, LDL: low-density lipoprotein, HDL: high-density lipoprotein, TSH: thyroid stimulant hormone.

**Table 2:** Transthoracic and strain echocardiographic findings of the patient groups.

	FBG 70–100 (*n* = 95)	FBG 100–125 (*n* = 53)	*p* Value
LV-GLS	−20.8 ± 4.1	−20.9 ± 2.6	0.803
LAS-r	52.3 ± 15	44.5 ± 10.7	0.001
LAS-cd	36.9 ± 11.7	28.4 ± 9.7	<0.001
LAS-ct	15.4 ± 9.4	16.1 ± 7.3	0.653
RV-FWSL	−29.5 ± 5.5	−27.9 ± 5.9	0.096
RV-GLS	−25.5 ± 6.9	−24.4 ± 5.2	0.297
LVEF (%)	65 ± 4.1	65.4 ± 4.2	0.594
LA diameter (cm)	3.1 ± 0.5	3.3 ± 0.4	0.036
RA diameter (cm)	3.3 ± 0.5	3.4 ± 0.5	0.050
LVEDD (cm)	4.6 ± 0.4	4.6 ± 0.5	0.925
LVESD (cm)	2.7 ± 0.4	2.6 ± 0.5	0.602
Mitral E (m/s)	0.8 ± 0.2	0.7 ± 0.2	0.037
Mitral A (m/s)	0.6 ± 0.1	0.6 ± 0.1	0.065
Mitral E/A ratio	1.5 ± 0.5	1.3 ± 0.4	0.005
Septal e′/a ratio	1.7 ± 0.5	1.2 ± 0.4	0.000
Lateral e′/a ratio	2.2 ± 0.9	1.8 ± 0.7	0.008
e′ (average)	12.9 ± 0.5	11.5 ± 2.4	0.004
E/e’ ratio	6.2 ± 1.4	6.6 ± 1.5	0.229
IVRT (ms)	82.7 ± 19.9	93.4 ± 22.6	0.005
DT (ms)	129.4 ± 33	134.8 ± 40	0.412
LA volume (mL/m^2^)	39.7 ± 16	48.1 ± 17.8	0.006
LVEDV (mL)	73.8 ± 20.1	80.5 ± 18.9	0.055
LVESV (mL)	26.2 ± 8.2	27.5 ± 7.1	0.326

**Abbreviations:** FBG: fasting blood glucose, LV-GLS: left ventricular global longitudinal strain, LAS-r: left atrium reservoir strain, LAS-cd: left atrium conduit strain, LAS-ct: left atrium contractile strain, RV-FWSL: right ventricular free wall longitudinal strain, RV-GLS: right ventricular global longitudinal strain, LVEDD: left ventricular end-diastolic diameter, LVESD: left ventricular end-systolic diameter, LA: left atrium, RA: right atrium, IVRT: isovolumetric relaxation time, DT: deceleration time, LVEDV: left ventricular end-diastolic volume, LVESV: left ventricular end-systolic volume.

**Table 3:** Determinants of left atrial reservoir strain by multivariate regression analysis.

Parameters	Univariate		Multivariate	
	r	*p*-Value	R^2^	*p*-Value
BMI (kg/m^2^)	−0.249	0.002		
Age (Year)	−0.214	0.008	0.500	0.15
LVEF (%)	−0.087	0.165		0.001
LV GLS	−0.235	0.004		0.002
Serum fasting glucose (mg/dL)	−0.268	0.001		<0.001

## Data Availability

The data presented in this study are available on request from the corresponding author. The data are not publicly available due to ethical reasons.
